# PilB from *Streptococcus sanguinis* is a bimodular type IV pilin with a direct role in adhesion

**DOI:** 10.1073/pnas.2102092118

**Published:** 2021-05-24

**Authors:** Claire Raynaud, Devon Sheppard, Jamie-Lee Berry, Ishwori Gurung, Vladimir Pelicic

**Affiliations:** ^a^Medical Research Council Centre for Molecular Bacteriology and Infection, Imperial College London, London SW7 2AZ, United Kingdom;; ^b^Laboratoire de Chimie Bactérienne, Aix-Marseille Université-CNRS (UMR 7283), Institut de Microbiologie de la Méditerranée, 13009 Marseille, France

**Keywords:** type IV pili, type IV filaments, type IV pilin, adhesion, von Willebrand factor A–like domain

## Abstract

Type IV pili (T4P) are functionally versatile filamentous nanomachines, nearly ubiquitous in prokaryotes. They are predominantly polymers of one major pilin but also contain minor pilins whose functions are often poorly defined and likely to be diverse. Here, we show that the minor pilin PilB from the T4P of *Streptococcus sanguinis* displays an unusual bimodular three-dimensional structure with a bulky von Willebrand factor A–like (vWA) module “grafted” onto a small pilin module via a short loop. Structural modeling suggests that PilB is only compatible with a localization at the tip of T4P. By performing a detailed functional analysis, we found that 1) the vWA module contains a canonical metal ion–dependent adhesion site, preferentially binding Mg^2+^ and Mn^2+^, 2) abolishing metal binding has no impact on the structure of PilB or piliation, 3) metal binding is important for *S. sanguinis* T4P–mediated twitching motility and adhesion to eukaryotic cells, and 4) the vWA module shows an intrinsic binding ability to several host proteins. These findings reveal an elegant yet simple evolutionary tinkering strategy to increase T4P functional versatility by grafting a functional module onto a pilin for presentation by the filaments. This strategy appears to have been extensively used by bacteria, in which modular pilins are widespread and exhibit an astonishing variety of architectures.

## Significance

Type IV pili (T4P) are functionally versatile filaments widespread in prokaryotes, composed of type IV pilins and assembled by conserved multiprotein machineries. It remains unclear how such rather simple filaments can be so versatile. Our structure/function analysis of PilB, a minor pilin of *Streptococcus sanguinis* T4P, offers an elegant explanation for this paradox. We show that PilB is a modular pilin with a bulky module “grafted” onto a small pilin module, which directly mediates adhesion of *S. sanguinis* to host cells/proteins. This evolutionary tinkering strategy appears to be prevalent in bacteria since a global analysis reveals that modular pilins are widespread and exhibit an astonishing variety of architectures.

Type IV pili (T4P) are functionally versatile filaments widespread in prokaryotes, implicated in a variety of functions such as adhesion, twitching motility, DNA uptake, etc (1). T4P are helical polymers consisting of type IV pilins, usually one major pilin and several minor (low abundance) ones, assembled by conserved multiprotein machineries. These defining features are shared by a superfamily of filamentous nanomachines known as type IV filaments (T4F) (1), ubiquitous in prokaryotes (2).

T4P have been intensively studied for decades in diderm bacteria because they play a central role in pathogenesis in important human pathogens (3). The following global picture of T4P biology has emerged from these studies. The pilus subunits, type IV pilins, are characterized by a short N-terminal sequence motif known as class III signal peptide, which consists of a hydrophilic leader peptide ending with a small residue (Gly or Ala), followed by a tract of 21 predominantly hydrophobic residues (4). This tract constitutes the N-terminal segment (α-1N) of an α-helix (α-1) of ∼50 residues, which is the universally conserved structural feature in type IV pilins. Usually, the α-1N helix protrudes from a globular head most often consisting of a β-sheet composed of several antiparallel β-strands, which gives pilins their characteristic “lollipop” shape (4). The hydrophilic leader peptide is then processed by a dedicated prepilin peptidase (5) after pilin translocation across the cytoplasmic membrane (CM) by the general secretory pathway (6, 7). Processed pilins remain embedded in the CM via their α-1N, generating a pool of subunits ready for polymerization. Filament assembly, which occurs from tip to base, is mediated at the CM by a complex multiprotein machinery (10 to 20 components) (1), centered on an integral membrane platform protein and a cytoplasmic extension ATPase (8). Recent cryogenic electron microscopy (cryo-EM) structures have revealed that T4P are right-handed helical polymers where pilins are held together by extensive interactions between their α-1N helices, which are partially melted and run approximately parallel to each other within the filament core (9, 10). One of the properties of T4P key for their functional versatility is their ability to retract, which has been best characterized for T4aP (where “a” denotes the subtype). In T4aP, retraction results from rapid filament depolymerization powered by the cytoplasmic retraction ATPase PilT (11), which generates important tensile forces (12, 13).

Studying T4P in monoderm bacteria represents a promising alternative research avenue (14). *Streptococcus sanguinis*, a commensal of the oral cavity that commonly causes life-threatening infective endocarditis (IE), has emerged as a monoderm model for deciphering T4P biology (15). Our comprehensive functional analysis of *S. sanguinis* T4P (16) revealed that they are canonical T4aP. Indeed, filaments are 1) assembled by a multiprotein machinery similar to diderm T4aP species but simpler with only 10 components, 2) retracted by a PilT ATPase, generating tensile forces similar to diderm species, and 3) powering intense twitching motility, leading to spreading zones around bacteria growing on plates, visible by the naked eye. Subsequently, we performed a global biochemical and structural analysis of *S. sanguinis* T4P (17), showing that 1) they are heteropolymers composed of two major pilins, PilE1 and PilE2, rather than one as usually seen, 2) the major pilins display classical type IV pilin three-dimensional (3D) structure, and 3) the filaments contain a low abundance of three minor pilins (PilA, PilB, and PilC), which are required for piliation.

The present study was prompted by a perplexing observation [i.e., the minor pilin PilB harbors a protein domain that has been extensively studied in eukaryotic proteins where it mediates adhesion to a variety of protein ligands (18)]. This suggested that PilB might be an adhesin, promoting T4P-mediated adhesion of *S. sanguinis* to host cells and proteins. Therefore, since both the molecular mechanisms of T4P-mediated adhesion and the exact role of minor pilins in T4P biology remain incompletely understood (1), we performed a structure/function analysis of PilB, which is reported here. This uncovered a widespread strategy for minor pilins to enhance the functional properties of T4P.

## Results

### PilB Displays a Modular Pilin Architecture.

**Fig. 1.**
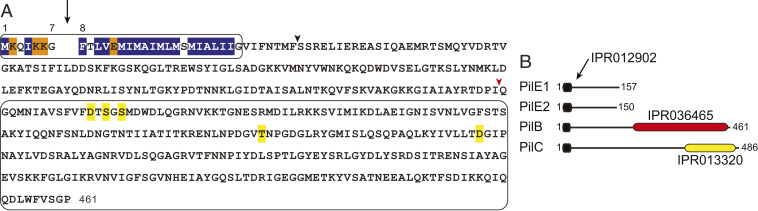
Bioinformatic analysis of PilB. (*A*) Relevant features of PilB from *S. sanguinis* 2908. The N-terminal class III signal peptide is boxed. The 7-aa long leader peptide contains mostly hydrophilic (shaded in orange) and neutral (no shading) residues, and it ends with a conserved Gly. This leader peptide is processed by the prepilin peptidase PilD, which is indicated by the vertical arrow, generating a protein of 454 residues (50.5 kDa). The processed protein starts with a tract of 21 predominantly hydrophobic residues (shaded in blue), which invariably form an extended α-helix that is the main assembly interface within filaments. The C-terminal vWA module (IPR036465) is boxed, with the conserved residues forming the MIDAS highlighted in yellow. Arrowheads indicate the proteins that were produced and purified in this study, consisting of either two modules (black arrowhead) or just the vWA module (red arrowhead). (*B*) Modular architectures of PilB and PilC minor pilins compared to the major pilins PilE1/PilE2. The proteins, from *S. sanguinis* 2908, have been drawn to scale. The black rounded rectangles correspond to the IPR012902 motif that is part of the class III signal peptide. The C-terminal domains in PilB and PilC are highlighted by colored rounded rectangles, vWA domain (red) and lectin domain (yellow).

PilB, one of the three minor pilins in *S. sanguinis* T4P (17), exhibits a canonical N-terminal class III signal peptide, the defining feature of type IV pilins (4). This sequence motif consists of a seven-residue leader peptide composed predominantly of hydrophilic and neutral amino acids (aa), ending with a Gly (Fig. 1*A*). This leader peptide, which is processed by the prepilin peptidase PilD (17), is followed by a stretch of 21 predominantly hydrophobic aa, except for a negatively charged Glu in position 5 (Fig. 1*A*). Processed PilB is unusually large for a pilin, with a predicted molecular mass of 50.5 kDa (Fig. 1*B*). For comparison, the two major pilins of *S. sanguinis* T4P, PilE1 and PilE2 (16), have typical pilin sizes of 14.7 and 14.1 kDa, respectively (Fig. 1*B*). The larger size of PilB is due to the presence of a C-terminal domain (Fig. 1*B*) readily detectable by bioinformatics, which belongs to the von Willebrand factor A–like domain superfamily (InterPro entry IPR036465). We will refer to this domain as vWA. The prototypical vWA domain is found in the von Willebrand factor (vWF), a human blood protein required for hemostasis (19), the physiological process that prevents/stops bleeding. vWA domains, which are found in more than 300,000 proteins in the three domains of life, have been extensively studied in eukaryotic proteins where they mediate adhesion to a variety of protein ligands (18). They have been much less studied in bacteria. Of note, the vWA domain in PilB is predicted to contain a metal coordination site known as MIDAS [metal ion–dependent adhesion site (20)] (Fig. 1*A*), which is important for ligand binding in several eukaryotic vWA-containing proteins (21).

The above type IV pilin architecture is unusual for two reasons. First, in contrast to classical pilins that consist only of a pilin module (4) defined by a short N-terminal IPR012902 motif (Fig. 1*B*), PilB apparently has an additional module. Second, the extra C-terminal module in PilB corresponds to a well-defined functional domain not specific to T4P biology, which has not been previously seen in pilins. Specifically, the second module—vWA—is often associated with adhesion to protein ligands (18, 21). This is what we call a modular architecture and why we refer to PilB as a modular pilin.

Taken together, these findings suggest that PilB is a modular pilin in which a functional module has been grafted during evolution onto a pilin moiety in order to promote T4P-mediated adhesion of *S. sanguinis* to protein ligands.

### Crystal Structure of PilB Reveals a Bimodular Pilin in which a Small Type IV Pilin Module Is Linked to a Bulky vWA Module via a Short Loop.

**Fig. 2.**
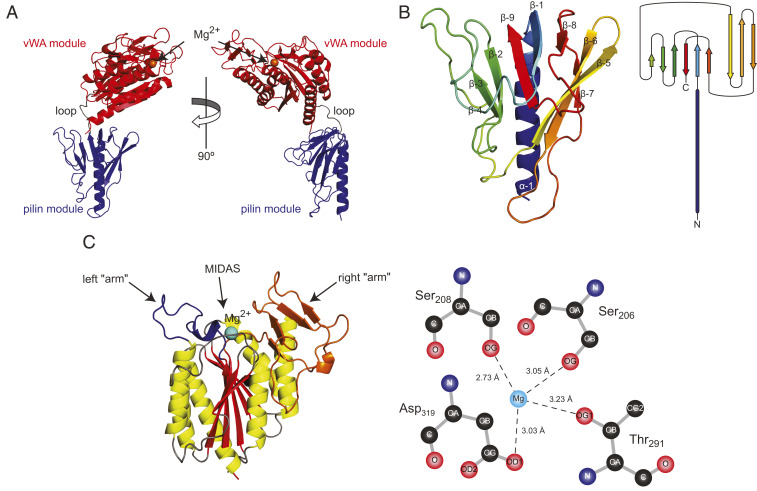
Crystal structure of PilB. (*A*) Orthogonal cartoon views of the PilB structure in which the two distinct modules have been highlighted in blue (pilin module) and red (vWA module), while the short loop connecting them is in gray. The orange sphere represents a magnesium ion. (*B*, *Left*) Close-up cartoon view of the pilin module colored in rainbow spectrum from blue (N terminus) to red (C terminus). (*B*, *Right*) Topology diagram of the pilin module structure. (*C*, *Left*) Close-up cartoon view of the vWA module in which the β-strands composing the central β-sheet are highlighted in red, while the surrounding α-helices are highlighted in yellow. The connecting loops are in gray, except for the two “arms” on top of the structure (colored in orange and blue), which surround the MIDAS. (*C*, *Right*) Diagram of the magnesium coordination by the conserved MIDAS residues. Coordinating oxygen atoms are shown, with dashed lines corresponding to hydrogen bonds.

High-resolution structural information was required to confirm that PilB is composed of two modules but also to understand how modular pilins are polymerized in the filaments and how they modulate T4P functionality. We therefore endeavored to solve the 3D structure of PilB by X-ray crystallography. To facilitate protein purification, we used a synthetic gene codon optimized for expression in *Escherichia coli* and produced a recombinant PilB protein in which the N-terminal 35 aa (encompassing the leader peptide and hydrophobic α-1N) (Fig. 1*A*) were replaced by a hexahistidine tag (6His) (17). This is a commonly used approach in the field to promote protein solubility since the truncation of α-1N has minimal structural impact on the rest of the protein (22). The resulting 48.4-kDa 6His-PilB protein was soluble and could be purified using a combination of affinity and gel-filtration chromatography. The protein readily crystallized in multiple conditions, and after optimizing the best diffracting crystals, we collected a complete dataset on crystals forming in the space group *P*6_1_ (Table 1). After phase determination, done using crystals produced in the presence of seleno-methionine (SeMet), we solved a high-resolution structure (2.26 Å) of native 6His-PilB. As can be seen in Fig. 2*A*, this structure reveals a clear bimodular architecture with a small pilin moiety (highlighted in blue) linked, via a short nine-residue loop (gray), to a bulky vWA moiety (in red).

**Table 1.** Crystal structures data collection and refinement statistics

Proteins	PilB	PilB_D319A_
(PDB)	(7B7P)	(7BA2)
Maximum resolution (Å)	2.26	3.00
Space group	*P*6_1_	*P*6_1_
Unit cell parameters		
a, b, c (Å)	124.38, 124.38, 140.74	120.95, 120.95, 151.25
α, β, ɣ (°)	90, 90, 120	90, 90, 120
Number of observations	329,504	208,399
Number of unique observations	57,372	25,101
R_merge_ (%)	6.2	16.6
I/σI	13.4	7.6
CC 1/2	0.999	0.998
Resolution range used for refinement	56.88 to 2.26	75.63 to 3.00
Completeness (%)	99.4	93.8
R factor (%)	20.5	24.1
Free R factor (%)	23.7	28.3
Ramachandran favored (%)	93.4	90.1
Ramachandran allowed (%)	6.4	8.6
Ramachandran outliers (%)	0.2	1.3
RMSD from ideal values		
bond length (Å)	0.008	0.005
bond angles (°)	0.974	1.273

While bioinformatics could only predict that the extreme N terminus of PilB corresponds to a class III signal peptide motif, our structure reveals that the first 180 residues of processed PilB clearly display a type IV pilin fold (4) and thus indeed correspond to a pilin module (Fig. 2*B*). The pilin module exhibits a long N-terminal α-helix packed against, not one β-sheet as usual, but two consecutive β-sheets consisting of six and three β-strands respectively, which together form the globular head of the pilin. The 432918 topology of the first β-sheet, that is, the order of the β-strands (Fig. 2*B*), is unusual since the β-strands are not contiguous along the protein sequence. Moreover, the last portion of this β-sheet forms a Ψ-loop (23) in which two antiparallel strands (β-8 and β-9) are linked via β-1 in between, connected to both of them by hydrogen bonds. This motif occurs rarely in proteins (23).

As for the vWA module (Fig. 2*C*), the structure strengthens the bioinformatic predictions. The vWA moiety of PilB adopts a canonical vWA fold (20, 24), with a central β-sheet (composed of five parallel and one antiparallel β-strands) surrounded on both sides by a series of α-helices (Fig. 2*C*). Consequently, the vWA module of PilB shows high structural similarity to many vWA-containing proteins with which it shares little sequence identity. For example, the vWA module of PilB is very similar to the third vWA domain of human vWF (25) (*SI Appendix*, Fig. S1), with an RMSD of 1.72 Å when the C_α_ atoms of the two structures are superposed. As in eukaryotic vWA-containing proteins (20, 24), PilB exhibits a MIDAS located on top of the central β-sheet (Fig. 2*C*). However, in contrast to these proteins, the MIDAS in PilB is flanked by two protruding “arms,” which is reminiscent of the RrgA adhesin from *Streptococcus pneumoniae* (26). While the first arm is perhaps unremarkable, the second folds into a four-stranded β-sheet (Fig. 2*C*). The MIDAS motif in PilB, which is formed by residues conserved in vWA-containing proteins, noncontiguous in the sequence (Fig. 1*A*) but in close proximity in the 3D structure (Fig. 2*C*), is functional since it coordinates a metal ion in the crystal. We have modeled the metal as Mg^+2^ because of its abundance in the growth medium and the high affinity of PilB for it (see Fig. 4). The Ser_206_, Ser_208_, Thr_291_, and Asp_319_ residues in the MIDAS motif (20) of PilB form direct hydrogen bonds with the metal through oxygen atoms (Fig. 2*C*), while two additional coordination sites are provided by water molecules.

**Fig. 3.**
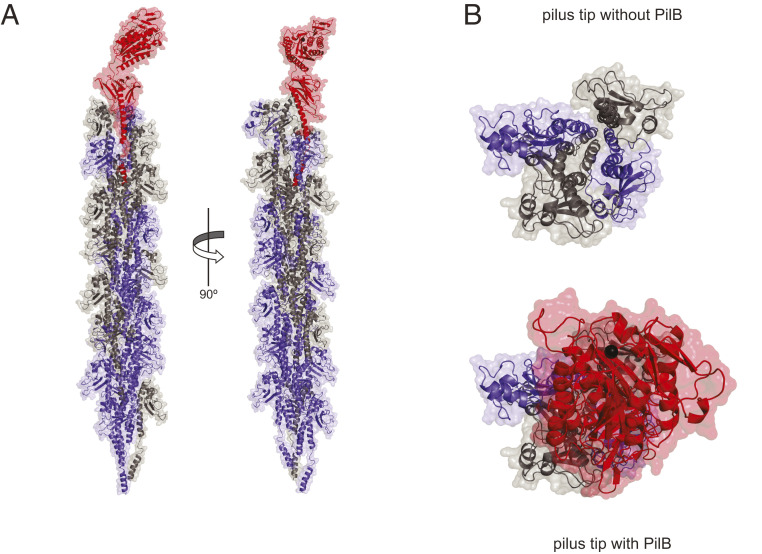
3D model of PilB in *S. sanguinis* T4P. (*A*) Packing of PilB (red) into *S. sanguinis* T4P, which is a right-handed helical heteropolymer of two major pilins PilE1 (blue) and PilE2 (gray). (*B*) View of the T4P tip capped by PilB or not.

An important biological implication of the PilB structure is that modular pilins, despite their large size, are likely to be polymerized into T4P in the same way as classical pilins (4) (i.e., via their N-terminal pilin module). We therefore tested by structural modeling whether PilB could pack into filaments. First, we produced a full-length 3D structural model of PilB including the missing α-1N (*SI Appendix*, Fig. S2), which was absent in the recombinant protein that we purified. Since a portion of α-1N in major pilins is melted during filament assembly, as observed in several T4aP cryo-EM structures (9, 10), the α-1N of PilB was modeled with a melted segment. This is consistent with the presence of the helix-breaking Gly residue in position 21 of α-1N (Fig. 1*A*). Then, we fitted this full-length PilB into a previously generated model of *S. sanguinis* T4P, a right-handed helical heteropolymer where major pilins PilE1/PilE2 are held together by interactions between their α-1N helices (Fig. 3*A*), which was based on the cryo-EM structure of *Neisseria meningitidis* T4P (9). Despite its unusual modular structure, PilB can be readily modeled into T4P, its pilin module establishing extensive hydrophobic interactions via its α-1N with the α-1N of neighboring major pilins (Fig. 3*A*). This suggests that PilB will assemble into filaments in the same way as classical pilins (9, 10). However, PilB can only be accommodated at the tip of the filaments because the bulky vWA module sits on top of the pilin module in the PilB structure and essentially prevents other pilin subunits from being modeled above it (Fig. 3*B*). Accordingly, when PilB is modeled in the body of the filament (*SI Appendix*, Fig. S3*A*), it exhibits important steric clashes with neighboring major pilins (*SI Appendix*, Fig. S3*B*).

Together, these structural findings show that PilB is a bimodular protein composed of two clearly distinct structural modules. The pilin module adopts a canonical type IV pilin fold (4), explaining how modular pilins are polymerized into T4P, most probably at their tip. The second module, which is linked to the end of the pilin module via a short loop, adopts a vWA fold (20, 24) with a clearly defined MIDAS that coordinates a metal. Since the vWA motif in many eukaryotic proteins is involved in adhesion to protein ligands (18, 21), our structure strengthens our working hypothesis that PilB might be an adhesin.

### Functional Analysis of the MIDAS in PilB Reveals that Metal Binding, although Structurally Dispensable, Is Important for T4P Functionality.

**Fig. 4.**
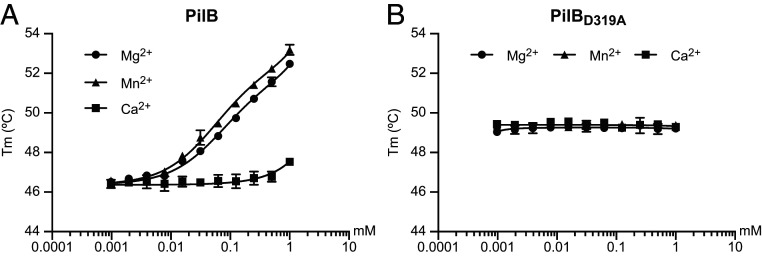
Metal binding by PilB. Purified PilB was incubated with increasing concentrations of divalent ions (Ca^2+^, Mg^2+^, and Mn^2+^), and binding was quantified by ThermoFluor. (*A*) Metal binding by PilB. (*B*) Metal binding by PilB_D319A_ with an inactive MIDAS. The results are the average ± SDs from three independent experiments.

Our PilB structure revealed that Mg^2+^, despite not being added during crystallization, is bound by the MIDAS. In eukaryotic proteins, the MIDAS sometimes coordinates Mn^2+^ as well (20, 24). We therefore tested the metal-binding specificity of the MIDAS in PilB using ThermoFluor. This fluorescent-based method, which measures changes in thermal denaturation temperature, is a commonly used approach for quantifying protein–ligand interactions (27). We determined the affinity of purified PilB for Mg^2+^, Mn^2+^, and Ca^2+^ (Fig. 4*A*). While no binding was detected to Ca^2+^, we found that PilB binds Mg^2+^ and Mn^2+^ efficiently in the micromolar range, with estimated *K*_d_ of 70 and 54 µM, respectively. To confirm that metal binding involves the MIDAS motif, we produced PilB_D319A_ in which the key MIDAS residue Asp_319_ (Fig. 2*C*) was changed into an Ala by site-directed mutagenesis. Binding assays performed with PilB_D319A_ showed that changing this single residue abolishes the metal-binding ability of PilB for both Mg^2+^ and Mn^2+^ (Fig. 4*B*). These findings show that the MIDAS in PilB is functional and preferentially binds Mg^2+^ and Mn^2+^

**Fig. 5.**
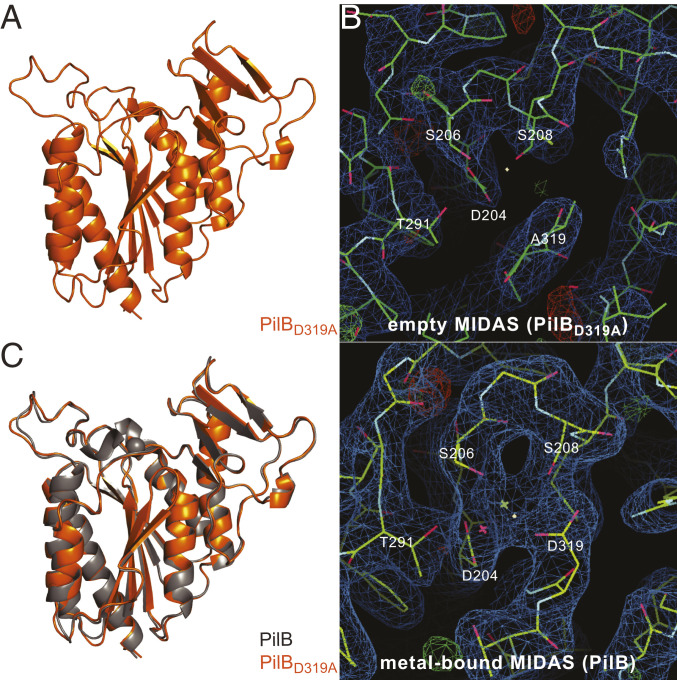
3D crystal structure of PilB_D319A_. (*A*) Close-up cartoon view of the vWA module in PilB_D319A_. (*B*) Comparison of electron density maps in the MIDAS pocket for the PilB_D319A_ (*Upper*) and PilB (*Lower*) structures. (*C*) Superposition of the vWA modules of PilB with bound Mg^2+^ (gray) and PilB_D319A_ (orange). The two structures superpose with an RMSD of 0.45 Å.

Next, to determine whether metal presence/absence might impact the 3D structure of PilB, we solved the structure of PilB_D319A_ by X-ray crystallography. The PilB_D319A_ protein readily crystallized in the same condition as the wild-type (WT) protein. We collected a complete dataset on crystals diffracting to a resolution of 3 Å (Table 1) and solved the structure of PilB_D319A_ (Fig. 5*A*) by molecular replacement. The structure of PilB_D319A_ (Fig. 5*A*) clearly shows that no metal is occupying the mutated MIDAS pocket on top of the central β-sheet (Fig. 5*B*), which is consistent with metal-binding assays. Although PilB_D319A_ resolution is significantly lower than WT, it nevertheless allows for meaningful structural comparison. When the vWA modules were compared, we found that they are essentially identical, the C_α_ atoms superposing onto each other (Fig. 5*C*) with an RMSD of merely 0.45 Å, including the two arms flanking the MIDAS pocket. This shows that metal binding by MIDAS has no detectable structural impact on PilB.

**Fig. 6.**
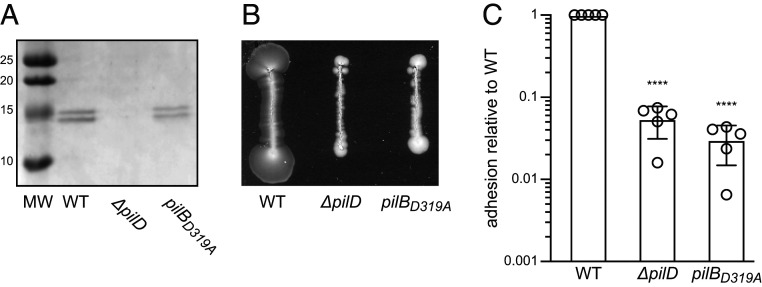
Phenotypic characterization of a *S. sanguinis* mutant expressing PilB_D319A_ with an inactive MIDAS. (*A*) Piliation was quantified by purifying T4P from cultures adjusted to the same OD_600_. Purified T4P (identical volumes were loaded in each lane) were separated by SDS-PAGE and stained with Coomassie blue. A molecular weight marker (MW) was run in the first lane. MWs are indicated in kDa. (*B*) Twitching motility was assessed by a macroscopic motility assay. Bacteria were streaked on plates, which were incubated several days at 37 °C in a humid atmosphere and then photographed. Twitching motility is characterized by spreading zones around colonies. (*C*) Adhesion of *S. sanguinis* to eukaryotic cells was quantified by incubating bacteria (MOI = 10) with CHO cells for 1 h. After removing nonadherent bacteria by several washes, bacteria adhering to cells were enumerated by performing CFU counts. The results are expressed as adhesion relative to WT (set to 1) and are the average ± SDs from five independent experiments. For statistical analysis, one-way ANOVA followed by Dunnett’s multiple comparison tests were performed (****P < 0.0001).

Next, we explored whether MIDAS-mediated metal binding by PilB is important for piliation and/or T4P-powered twitching motility, both of which were previously shown to be abolished in a *ΔpilB* mutant (16). We therefore constructed an unmarked *S. sanguinis* mutant in which the endogenous *pilB* gene was altered by site-directed mutagenesis to produce PilB_D319A_. We first tested whether the *pilB*_*D319A*_ mutant retains the ability to assemble T4P using filament purification (16). As can be seen in Fig. 6*A*, in which purified T4P were separated by sodium dodecyl sulphate–polyacrylamide gel electrophoresis (SDS-PAGE) and stained with Coomassie blue, the *pilB*_*D319A*_ mutant is piliated. This is evidenced by the presence of the two bands corresponding to major pilins PilE1 and PilE2, which are absent in a nonpiliated *ΔpilD* control (Fig. 6*A*). Moreover, the amount of T4P that can be purified from the *pilB*_*D319A*_ mutant and WT strain appear comparable. We then tested whether the pili in the *pilB*_*D319A*_ mutant are able to mediate twitching motility (16). For the WT strain, twitching motility is evidenced by spreading zones around bacteria grown on agar (Fig. 6*B*). Spreading zones were absent for the *pilB*_*D319A*_ mutant (Fig. 6*B*). This shows that the MIDAS-mediated metal-binding ability of PilB, while dispensable for piliation, is important for T4P-mediated twitching motility.

Together, these findings show that the MIDAS in PilB is a functional metal-binding site, dispensable for piliation and protein folding but essential for T4P functionality.

### T4P-Mediated Adhesion to Eukaryotic Cells Requires PilB, which Specifically Binds Several Human Proteins.

Since vWA is involved in adhesion in many eukaryotic proteins (18, 21), our original hypothesis was that PilB might mediate *S. sanguinis* adhesion to host cells and/or proteins, which we aimed to test next. First, we determined whether T4P might be involved in its well-known ability of *S. sanguinis* to adhere to host cells (28). After testing a few eukaryotic cell lines, we opted for Chinese hamster ovary (CHO) cells because the WT strain adheres very efficiently to them. When CHO cells were infected by *S. sanguinis* at a multiplicity of infection (MOI) of 10, 31.6 ± 9.1% of the bacterial inoculum adhered to the cells (Fig. 6*C*). In contrast, a nonpiliated *ΔpilD* mutant showed a significantly reduced adhesion, with an 18-fold decrease relative to the WT (Fig. 6*C*). Next, we tested our original assumption that PilB might be an adhesin by quantifying the adhesion of the *pilB*_*D319A*_ mutant. As can be seen in Fig. 6*C*, although the *pilB*_*D319A*_ mutant is piliated, its adhesion to CHO cells is dramatically impaired, with a 33-fold decrease when compared to the WT. These findings show that *S. sanguinis* T4P are multifunctional filaments important for adhesion to eukaryotic cells and that PilB plays an important role.

**Fig. 7.**
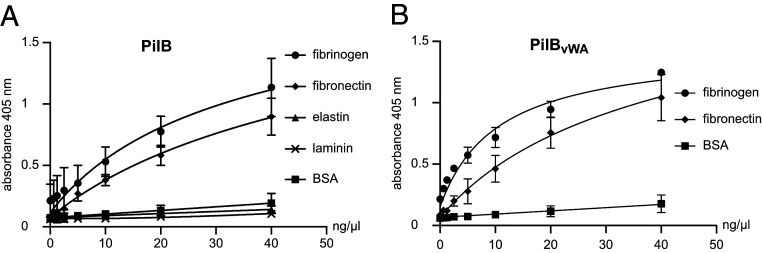
Dose-dependent binding of PilB to various protein ligands. Increasing concentrations of purified PilB was added to constant concentrations of immobilized putative ligands, and binding was quantified by ELISA. BSA served as negative control. The results are the average ± SDs from at least three independent experiments. (*A*) Binding of PilB to fibrinogen, fibronectin, elastin, and laminin. (*B*) Binding of PilB_vWA_, consisting only of the vWA module, to fibrinogen and fibronectin.

Since the vWA domain in multiple eukaryotic proteins has been shown to mediate cell–extracellular matrix (ECM) interactions (21), we reasoned that PilB might recognize similar ligands because it exhibits a canonical vWA module (*SI Appendix*, Fig. S1). We tested this hypothesis by performing binding assays with purified PilB using enzyme-linked immunosorbent assay (ELISA). In brief, we coated 96-well plates with selected putative ligands, added serial dilutions of purified 6His-PilB, and detected binding using an anti-6His antibody. We tested binding to fibrinogen and the ECM proteins fibronectin, elastin, and laminin. While PilB exhibits no binding to bovine serum albumin (BSA) that was used as a negative control (Fig. 7*A*), we observed dose-dependent binding to fibronectin and fibrinogen but not to the other ECM proteins that were tested (elastin and laminin). Specific binding to fibronectin and fibrinogen was in the high nanomolar range, with calculated *K*_d_ of 494 and 865 nM, respectively (Fig. 7*A*). Under these in vitro experimental conditions, metal coordination by the MIDAS is dispensable for binding to fibronectin and fibrinogen since PilB_D319A_ binds these ligands as well as PilB (*SI Appendix*, Fig. S4). Finally, to confirm the prediction that binding of PilB to the above ligands is mediated by its vWA module, we produced and purified PilB_vWA_ corresponding only to the vWA module (Fig. 1*A*). We found that PilB_vWA_ binds to fibronectin and fibrinogen (Fig. 7*B*), with calculated *K*_d_ of 997 and 337 nM, respectively, which were comparable to PilB. These findings confirm that the adhesive ability of PilB is due to its vWA module.

Taken together, these findings show that *S. sanguinis* T4P are multifunctional filaments mediating adhesion to eukaryotic cells and that PilB is a bona fide adhesin using its vWA module to bind several human protein ligands it shares with eukaryotic vWA-containing proteins.

### Pilins with Modular Architectures Are Widespread in Bacteria.

**Fig. 8.**
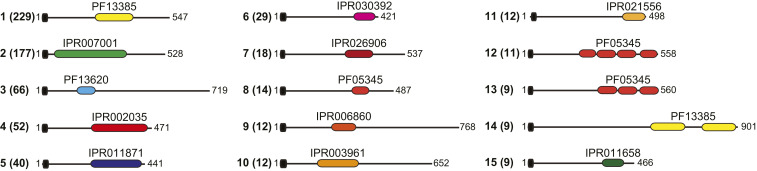
Global distribution of modular pilins. The 15 most widespread modular pilin architectures in the InterPro database are presented. The numbers in parenthesis represent the number of proteins displaying that architecture. The representative proteins depicted, drawn to scale, are from the following species: 1) *Candidatus* Magasanikbacteria (UniProtKB/TrEMBL protein A0A0G0IU57), 2) *Photorhabdus luminescens* (A0A022PI42), 3) *Desulfuribacillus stibiiarsenatis* (A0A1E5L295), 4) *Candidatus* Falkowbacteria (A0A1J4TDE2), 5) *Candidatus* Wolfebacteria (A0A0G1WFE5), 6) Clostridiales bacterium (A0A101H8M7), 7) *Corynebacterium glutamicum* (A0A1Q6BQB1), 8) *Thermosulfidibacter takaii* (A0A0S3QUH2), 9) *Candidatus* Gracilibacteria (A0A1J5F7A7), 10) *Candidatus* Saccharibacteria (A0A1Q3NLQ9), 11) Planctomycetes bacterium (A0A1G2ZHU9), 12) *Actinoplanes awajinensis* subsp. *mycoplanecinus* (A0A101J7V4), 13) *Actinoplanes derwentensis* (A0A1H2D7E9), 14) Desulfobacterales bacterium (A0A1V1WSE4), and 15) Parcubacteria group bacterium (A0A2D6FLV5).

PilB orthologs are ubiquitous in *S. sanguinis*, which also produces a second modular pilin PilC (17), where the extra module belongs to the concanavalin A–like lectin/glucanase domain superfamily (IPR013320) (Fig. 1*B*). We wondered how widespread and diverse modular pilins might be. We therefore searched the InterPro database (29) for all the proteins with an N-terminal IPR012902 domain, which also contain an extra domain not specific to T4P biology. This showed that modular pilins are 1) widespread with more than 1,200 proteins displaying such architecture (Dataset S1), 2) present both in monoderm and diderm species, and 3) highly diverse, with as many as 264 different architectures detected. Although a bimodular architecture is the most prevalent, there are modular pilins with multiple additional domains, the most extreme case being an 860-residue protein from *Candidatus* Falkowbacteria, with 12 copies of the IPR013211 motif of unknown function (Dataset S1). A closer inspection of the 15 most frequent modular pilin architectures offers a glimpse of their diversity (Fig. 8). While in many of these proteins the extra domain has no clear function (IPR007001, IPR011871, IPR026906, PF05345, IPR006860, IPR003961, and IPR021556), for others a function can be predicted. These functions include 1) binding to carbohydrates via PF13385 (that overlaps with the IPR013320 lectin domain superfamily), PF13620 (carbohydrate-binding–like fold), or IPR011658 (PA14 carbohydrate-binding domain), 2) binding to proteins via IPR002035 (that overlaps with the IPR036465 vWA domain superfamily), or even 3) peptidase activity via IPR030392. These findings suggest that the rather simple modular design strategy—where a functional module is grafted during evolution onto a pilin—appears to have been used often during evolution both by monoderm and diderm bacteria and is expected to increase the functional versatility of T4P.

## Discussion

T4F are an important research topic because of their virtual ubiquity in prokaryotes and their ability to mediate several key biological processes (1). Furthermore, the molecular mechanisms of T4F-mediated functions and the exact role of minor pilins remain incompletely understood. Therefore, in this report we focused on T4aP—the prototypical T4F (1)—in the recently established monoderm model *S. sanguinis* (15) and performed a structure/function analysis of the unusual minor pilin PilB, which we predicted might play a role in T4P-mediated adhesion. This led to several notable findings discussed below and confirmed predictions that the study of T4P in monoderms has the potential to shine new light on these filaments (14, 15).

The first important finding in this study is that modular type IV pilins—T4F subunits in which an N-terminal pilin module is fused via a short linker to a functional module with a direct role in a T4F-mediated function—are widespread and extremely diverse. Modular pilins are likely to be tip exposed in the filaments because of their peculiar architecture. However, a location in the body of the filament cannot be excluded provided that the linker is flexible enough. While previous 3D structures of a few large minor pilins suggest that they are modular pilins, their second modules do not correspond to protein domains readily identifiable by available bioinformatic tools. For example, in CofB from enterotoxigenic *E. coli* (ETEC) T4bP, there are two additional structural domains linked to the C terminus of the pilin module by a flexible linker, a β-repeat domain followed by a β-sandwich domain (30). CofB, which forms a trimer predicted to be exposed at the tip of ETEC T4bP (31), appears to be an adapter for a secreted protein CofJ (32) that has a direct role in adhesion. TcpB, from *Vibrio cholerae* T4bP toxin-coregulated pilus, is a tip-located minor pilin forming trimers with a structure very similar to CofB (33), which probably has a similar function. Incidentally, CofB is also the receptor for the bacteriophage CTXϕ (33). In ComZ from *Thermus thermophilus* T4aP, the additional structural domain is a large β-solenoid inserted not at the end of the pilin module but into the β-sheet (34), and is thought to mediate binding of extracellular DNA during transformation (34). This modular architecture is not restricted to T4P as it is also observed for a minor pilin from another T4F, GspK from type II secretion systems (T2SS) (35). In GspK, the additional structural domain is an α-domain of unclear function inserted into the β-sheet of the pilin module. GspK has been proposed to be at the tip of T2SS pseudopili, together with two other nonmodular minor pilins (GspI and GspJ) with which it interacts to form a heterotrimer (35). These examples suggest that we have probably underestimated the global distribution of modular pilins, which are likely to be much more widespread because in many of them, the additional modules are not yet defined by protein signatures in the databases. However, what is clear from our global analysis is that the functions associated with these modular pilins are potentially extremely diverse. Although a “common theme” appears to be the interaction of T4F with a variety of ligands—including proteins (via vWA in PilB, and the β-repeat/β-sandwich module in CofB), carbohydrates (via a variety of lectin domains including the concanavalin A–like lectin/glucanase domain in PilC), or DNA (the role of the β-solenoid module in ComZ)—other previously unreported T4F functions are possible. This is suggested by the modular architectures IPR012902-IPR030392 or IPR012902-IPR011493, in which the second module is a predicted peptidase belonging to S74 and M26 families, respectively.

The functional characterization of the vWA module in PilB including its MIDAS, showing that it is a bona fide adhesin, is another significant achievement of this study. First, the vWA domain, which is ubiquitous in the three domains of life and has been extensively studied in eukaryotes (18, 21), has been much less studied in bacteria. Second, T4P-mediated adhesion remains among the least understood T4P functions (1). Our functional analysis of the vWA module in PilB significantly extends what was known for prokaryotic vWA-containing proteins and highlights important similarities and differences with eukaryotic vWA-containing proteins. Our 3D structure shows that the vWA module in PilB exhibits striking similarity to the vWA domain in eukaryotic proteins (20, 24), with a canonical MIDAS coordinating a metal. The main difference is that the MIDAS in PilB is flanked by two protruding arms, similar to what has been described for RrgA from *S. pneumoniae* (26). Interestingly, RrgA is a subunit with intrinsic adhesive properties (36) of sortase-assembled pili in monoderms (37), which are unrelated to T4P. The parallel between RrgA and PilB denotes a case of convergent evolution in which two unrelated types of pili have evolved a similar strategy to mediate adhesion. Testing metal binding by the MIDAS in PilB, which was previously done only for eukaryotic vWA-containing proteins (38), highlights important similarities. MIDAS shows no significant binding to Ca^2+^ and a slight preference for Mn^2+^ over Mg^2+^, although the difference in affinity is much smaller than in eukaryotic proteins (38). Metal binding can be abolished by altering the MIDAS motif, which has no impact on PilB structure (38, 39). Abolishing metal binding has no detectable effect on piliation, which is analogous to what has been reported for vWA-containing adhesins of sortase-assembled pili (40, 41), but it impairs T4P-mediated twitching motility. It is unclear at this stage whether the lack of motility of the *pilB*_*D319A*_ mutant is due to reduced T4P-mediated adhesion to the agar, which would be consistent with PilB role in adhesion, or to impaired filament retraction (11). We also provide evidence that the vWA module of PilB binds several human protein ligands that it shares with eukaryotic vWA-containing proteins such as integrins and/or vWF (19, 21). However, unlike in these proteins where binding is often impaired when the MIDAS is inactivated (20), binding to fibronectin and fibrinogen is unaffected in a PilB_D319A_ mutant. This either suggests that the MIDAS is not implicated in binding these specific ligands, which has been described for vWF binding to collagen (25), or that our in vitro binding assay is not sensitive enough to detect subtle but significant differences in binding.

The finding that PilB plays a key role in adhesion of *S. sanguinis* to host cells and structures via its vWA module has implications for the pathogenesis of this species in particular and for our understanding of T4P-mediated adhesion in general. Our findings are consistent with the possibility that PilB-mediated adhesion to host proteins might play a role in IE (42), a life-threatening infection often caused by *S. sanguinis*. Indeed, during IE, bacteria that have gained access to the bloodstream adhere to preexisting sites of valvular damage where ECM proteins are exposed, and a blood clot is present containing large amounts of platelets, fibrinogen/fibrin, and fibronectin (43). Our finding that PilB adheres directly to two of these proteins, but additional ligands cannot be excluded, suggests that PilB might be important at this early stage in IE, which could be tested in future studies. Our findings, which arguably make PilB the best-characterized T4P adhesin alongside PilC/PilY1 found in diderm T4aP (44–48), have general implications for our understanding of T4P-mediated adhesion. The vWA module in PilB, which is most likely exposed at the pilus tip, is ideally placed to maximize bacterial adhesion to host protein receptors. T4P spring-like properties—gonococcal T4P can be stretched three times their length (49)–are expected to help bacteria that are bound via a tip-located adhesin to withstand adverse forces in their particular environment (e.g., blood flow in a heart valve). This is likely to apply to other modular pilins as well, which harbor different modules predicted to function in adhesion. The parallel with the best-characterized T4P adhesin PilC/PilY1 is obvious. This protein, which is not a pilin, is an adhesin that has been proposed to be presented at the T4P tip (45) via its interaction with a tip-located complex of four widely conserved minor pilins (50). All PilC/PilY1 have in common a C-terminal IPR008707 β-propeller domain while their N-termini are different (51). Since this is analogous to the situation with modular pilins, we wondered whether it could be an indication of a modular design for PilC/PilY1. This indeed seems to be the case since a search of the InterPro database (29) for all the proteins with an IPR008707 domain shows that 68 different PilC/PilY1 modular architectures are detected (Dataset S2). Strikingly, many of the N-terminal modules in PilC/PilY1 are shared with modular pilins, including vWA that was identified in PilY1 from *Pseudomonas aeruginosa* (52). These observations suggest that the same tinkering strategy has been used both by pilins and PilC/PilY1 to increase the functional versatility of T4P. In both instances, a “carrier” module for presentation at the tip of the filaments (either a pilin, or an IPR008707 domain that interacts with a tip-located complex of minor pilins) has been fused to variety of “effector” modules directly involved in diverse functions.

In conclusion, by performing a detailed structure/function analysis of the minor pilin PilB from *S. sanguinis*, we have shed light on several aspects of T4P biology. Our findings are not only of relevance for *S. sanguinis*, most notably for colonization of its human host, they have general implications for T4F by uncovering a prevalent strategy used by these widespread filamentous nanomachines to promote their well-known exceptional functional versatility (1). The resulting conceptual framework paves the way for further investigations, which will further improve our understanding of these fascinating filaments.

## Materials and Methods

### Strains and Growth Conditions.

Strains and plasmids used in this study are listed in *SI Appendix*, Table S1. For cloning, we used *E. coli* DH5-α. For protein purification, we used *E. coli* BL21(DE3) or *E. coli* BL21 B834(DE3) (*SI Appendix*). Chemically competent *E. coli* cells were prepared as described (53). DNA manipulations were done using standard molecular biology techniques (54). PCR were done using high-fidelity DNA polymerases (Agilent). Primers used in this study are listed in *SI Appendix*, Table S2. The pET-28b (Novagen) derivative, pET28-*pilB*_*36461*_ for expressing 6His-PilB_36-461_ was described previously (17). In this plasmid, the portion of a synthetic *pilB* gene codon optimized for expression in *E. coli*, encoding the soluble portion of PilB, was fused to a noncleavable N-terminal 6His tag. Similarly, we constructed pET28-*pilB*_*192461*_ for expressing PilB_vWA_. To construct pET28-*pilB*_*D319A*_ for expressing 6His-PilB_D319A_, we introduced a missense mutation in pET28-*pilB*_*36461*_ using QuikChange site-directed mutagenesis (Agilent).

The WT *S. sanguinis* 2908 strain and deletion mutants (*ΔpilD* and *ΔpilB*) were described previously (16). *S. sanguinis* genomic DNA was prepared from overnight (O/N) liquid cultures using the XIT Genomic DNA from Gram-Positive Bacteria kit (G-Biosciences). Strain 2908, which is naturally competent, was transformed as described (16, 55). The unmarked *S. sanguinis pilB*_*D319A*_ mutant was constructed using a previously described two-step gene editing strategy (55) (*SI Appendix*).

### Protein Purification.

To purify native PilB, PilB_D319A_, and PilB_vWA_ proteins, the corresponding pET-28b derivatives were transformed in *E. coli* BL21(DE3). Expression and purification are detailed in *SI Appendix*. To purify SeMet-labeled PilB for phasing, the corresponding pET-28b derivative was transformed in *E. coli* BL21 B834(DE3). Expression and purification are detailed in *SI Appendix*

### Crystallization and Structure Determination.

Purified proteins in 50 mM Hepes (pH 7.4) and 200 mM NaCl were concentrated to 50 mg/mL and tested for crystallization using sitting-drop vapor diffusion, with 100-nL drops of protein solution and mother liquor. We tested a range of commercially available kits (Molecular Dimensions, Hampton Research, and Rigaku Reagents), which yielded a number of hits, mainly in high-salt conditions. Crystallization conditions were optimized to yield larger and better diffracting crystals. All data were collected and processed using the Diamond Light Source beamline i03 and integrated in *P*6_1_ using the 3dii pipeline in *xia*2 (56). Initial molecular replacement was performed with Phaser (57) on the 2.26-Å resolution PilB dataset using a low-resolution partial model produced from the SeMet data using autoSHARP (58). Manual building in Coot (59) was performed on the high-resolution dataset, and the full model was then used for molecular replacement in the low-resolution datasets. All structures were produced using Coot and phenix.refine (60) and validated using MolProbity (61).

### Assaying Metal Binding by Purified PilB.

The metal-binding specificity of PilB was tested using ThermoFluor, a fluorescent-based method measuring changes in thermal denaturation temperature (27). Assays were done in a 96-well plate (Applied Biosystems) format. In the wells, we added to a final volume of 40 µL 1) 0 to 1 mM range of concentrations of MgCl_2_, MnCl_2_, and CaCl_2_, 2) 20 µM purified PilB or PilB_D319A_, and 3) 1/5,000 dilution of SYBR Orange (Thermo Fisher Scientific). Plates were then analyzed using a temperature gradient, from 25 to 99 °C, on a StepOnePlus RT-qPCR machine (Applied Biosystems). The data were exported in MATLAB and analyzed in GraphPad. Analyses were performed with Prism (GraphPad Software). *K*_d_ were calculated using nonlinear regression fits, applying saturation binding equation (One site − Total and nonspecific binding) using Ca^2+^ as nonspecific binding control.

### Assaying Protein–Ligand Binding by Purified PilB.

Binding of PilB, PilB_vWA_, and PilB_D319A_ to a variety of eukaryotic proteins was tested by ELISA as follows. Putative ligand proteins (elastin from human skin, fibrinogen from human plasma, laminin from human placenta, and fibronectin from human plasma) (all from Sigma) were resuspended in carbonate-bicarbonate buffer (Sigma) at 5 µg/mL. A total of 50 µL was dispatched into the wells of MaxiSorp plates and adsorbed O/N at 4 °C. Wells were washed three times with phosphate-buffered saline (PBS) (Gibco) and blocked during 1 h with 3% BSA (Probumin) or 1% gelatin (Sigma) in PBS. After washing with PBST (PBS containing 0.05% Tween 20), serial twofold dilutions of PilB (from 40 to 0.625 µg/mL) were added to the wells and incubated for 2 h at 37 °C. After five washes with PBST, we added 50 µL anti-6His RTM antibody (Abcam) at 1/500 dilution in PBS and incubated for 1 h at room temperature (RT). After five washes with PBST, we added 50 µL Amersham ECL anti-rabbit IgG horseradish peroxidase–linked whole antibody (GE Healthcare) at 1/500 dilution in PBS and incubated for 1 h at RT. After five washes with PBST, we added 100 µL/well of 3,3′,5,5′-Tetramethylbenzidine solution (Thermo Scientific) and incubated the plates during 20 min at RT in the dark. Finally, we stopped the reaction by adding 100 µL/well of 0.18 M sulfuric acid before reading the plates at 450 nm using a plate reader. Analyses were performed with Prism (GraphPad Software). *K*_d_ were calculated using nonlinear regression fits, applying saturation binding equation (One site − Total and nonspecific binding) using BSA or gelatin as nonspecific binding control.

### Assaying Piliation of *S. sanguinis*.

*S. sanguinis* T4P were purified as described (16, 17) (*SI Appendix*). Pili were resuspended in pilus buffer, separated by SDS-PAGE, before gels were stained with Bio-Safe Coomassie (Bio-Rad).

### Assaying Twitching Motility of *S. sanguinis*.

Twitching motility was assessed on agar plates as described (16) (*SI Appendix*). Plates were photographed using an Epson Perfection V700 photo scanner.

### Assaying Adhesion of *S. sanguinis* to Eukaryotic Cells.

We tested adhesion of *S. sanguinis* to CHO cells (Public Health England) as follows. Cells were replicated in flasks in Dulbecco’s Modified Eagle Medium (Gibco) containing 1× minimum essential medium nonessential aa mix (Gibco) and 5% FBS (Gibco) and seeded at 100,000 cells/cm^2^ in 24-well plates, which were incubated O/N at 37 °C in the presence of 5% CO_2_. The next day, cell monolayers were gently rinsed with PBS and infected at an MOI of 10 with bacteria grown in Todd Hewitt broth (TH). In brief, bacteria were grown for a few hours to optical density (OD)_600_ 0.5 units, adjusted at the same OD, pelleted by centrifugation at 1,100 *g* during 10 min, and resuspended in PBS. Bacteria in the inoculum were quantified by performing colony-forming unit (CFU) counts on TH plates. After 1 h of infection at 37 °C, cell monolayers were gently rinsed four times with PBS before cells with adherent bacteria were scraped in distilled water. Adherent bacteria were then quantified by performing CFU counts. Statistical analyses were performed with Prism. Comparisons were done by one-way ANOVA, followed by Dunnett’s multiple comparison tests. An adjusted *P* < 0.05 was considered significant (*P < 0.05, **P < 0.01, ***P < 0.001, ****P < 0.0001).

### Bioinformatics.

Protein sequences were routinely analyzed using DNA Strider (62). Prediction of protein domains, their global distribution, and associated architectures was done by using InterProScan (29) to interrogate the InterPro database. This database was also used to download all the protein entries discussed in this paper. Molecular visualization of protein 3D structures was done using PyMOL (Schrödinger). The PDBsum Generate (63) server was used to provide at-a-glance overviews—secondary structure, topology diagram, protein motifs, and schematic diagram of metal–protein interactions—of the 3D structures determined during this work. The Dali (64) server was used for comparing protein structures in 3D. Protein 3D structures were downloaded from the Research Collaboratory for Structural Bioinformatics Protein Data Bank (PDB) server. The 3d-SS (65) server was used to superpose 3D protein structures with the STAMP algorithm (66).

The cryo-EM structure of *N. meningitidis* T4P (PDB 5KUA) (9) was used to model, using SWISS-MODEL (67), the N-terminal helices of PilE1, PilE2, and PilB within the filaments. Coot and PyMOL were then used to place the full-length structures within the T4P model.

## Supplementary Material

Supplementary File

Supplementary File

Supplementary File

## Data Availability

3D structures have been deposited in the PDB and are available under accession codes 7B7P (PilB) and 7BA2 (PilB_D319A_). All the datasets generated during this study are included in this paper and *SI Appendix*
