# Supplementary material for: PilB from Streptococcus sanguinis is a bimodular type IV pilin with a direct role in adhesion
Source: Proc Natl Acad Sci U S A. 2021 May 24;118(22):e2102092118. doi: 10.1073/pnas.2102092118 (PMC8179133; doi:10.1073/pnas.2102092118)
Supplement: Supplementary File [file pnas.2102092118.sapp.pdf]

PilB from *Streptococcus sanguinis* is a bimodular type IV pilin with a direct role in adhesion

Claire Raynaud[a,1], Devon Sheppard[a,1], Jamie-Lee Berry[a], Ishwori Gurung[a], Vladimir Pelicic[a,b,2]

[a]MRC Centre for Molecular Bacteriology and Infection, Imperial College London, London SW7 2AZ, United Kingdom

[b]Laboratoire de Chimie Bactérienne, Aix-Marseille Université-CNRS (UMR7283), Institut de Microbiologie de la Méditerranée, 13009 Marseille, France

[1]C.R. and D.S. contributed equally to this work

[2]To whom correspondence may be addressed. Email: vladimir.pelicic@inserm.fr

Materials and methods

Strains and growth conditions

E. coli was grown in liquid or solid Lysogenic Broth (LB) (Difco) containing, when required, 100 µg/ml spectinomycin or 50 µg/ml kanamycin (both from Sigma). For purification of protein labelled with seleno-methionine (SeMet), bacteria were grown in chemically defined medium (CDM) supplemented with 20 mg/ml SeMet (Sigma).

S. sanguinis was grown on plates containing Todd Hewitt (TH) broth (Difco) and 1 % agar (Difco), incubated at 37ºC in anaerobic jars (Oxoid) under anaerobic conditions generated using Anaerogen sachets (Oxoid) (1). Liquid cultures were grown statically under aerobic conditions in THT, *i.e.*, TH broth containing 0.05 % tween 80 (Merck) to limit bacterial clumping(1). When required, 500 µg/ml kanamycin was used for selection and 15 mM *p*-Cl-Phe (Sigma) for counterselection (2). To construct the unmarked *S. sanguinis* $pilB_{D319A}$ mutant, we replaced the gene in the WT by a promoterless *pheS*aphA-3* double cassette, which confers sensitivity to *p*-Cl-Phe and resistance to kanamycin (2). To do this, we fused by splicing PCR the upstream and downstream regions flanking *pilB* to *pheS*aphA-3*, directly transformed the PCR product into the WT, and selected allelic exchange mutants on kanamycin plates. Allelic exchange was confirmed by PCR. In the second step, we replaced the *pheS*aphA-3* double cassette in this primary mutant by allelic exchange, with an unmarked $pilB_{D319A}$ mutation. To do this, we first constructed the missense mutation by site-directed mutagenesis, using as a template a pCR8/GW/TOPO (Invitrogen) derivative in which the WT gene was cloned. Then, the PCR product was directly transformed into the primary mutant, with plating on *p*-Cl-Phe-containing plates. Markerless allelic exchange mutants, which are the only one sensitive to kanamycin, were identified by re-streaking colonies on TH plates with and without antibiotic.

Protein purification

To purify native PilB, $PilB_{D319A}$ and $PilB_{vWA}$ proteins, the corresponding pET-28b derivatives were transformed in *E. coli* BL21(DE3). Transformants were grown O/N at 37ºC in liquid LB with kanamycin. The next day, this culture was diluted (1/500) in 1 l of the same medium and grown at 37ºC to an OD_{600} of 0.4-0.6. Proteins were purified as described (3).The temperature was then set to 16ºC, the culture allowed to cool for 30 min, before protein expression was induced O/N by adding 0.5 mM IPTG (Merck). The next day, cells were harvested by centrifugation at 8,000 *g* for 20 min and subjected to one cycle of -80ºC freeze/thaw in binding buffer (50 mM HEPES pH 7.4, 200 mM NaCl, 15 mM imidazole), to which we added SIGMAFAST EDTA-free protease inhibitor cocktail (Sigma). Cells were disrupted by repeated cycles of sonication, *i.e.*, pulses of 5 sec on and 5 sec off during 3-5 min, until the cell suspension was visibly less viscous. The cell lysate was then centrifuged for 30 min at 17,000 *g* to remove cell debris. The clarified lysate was first affinity-purified on an ÄKTA Purifier using His-Trap HP columns (GE Healthcare) and eluted with elution buffer (50 mM HEPES pH 7.4, 200 mM NaCl, 300 mM imidazole). Affinity-purified proteins were further purified, and simultaneously buffer-exchanged into (50 mM HEPES pH 7.4, 200 mM NaCl), by gel-filtration chromatography on an ÄKTA Purifier using a Superdex 75 10/300 GL column (GE Healthcare). Protein concentration was quantified spectrophotometrically using a NanoDrop Lite (Thermo Fisher Scientific).

To purify SeMet-labelled PilB for phasing, the corresponding pET-28b derivative was transformed in *E. coli* BL21 B834(DE3). Transformants were grown at 37ºC in liquid LB with kanamycin, until OD_{600} reached 0.6-0.7. Next, the cells were pelleted at 8,000 *g* for 5 min, and washed twice with 2 ml of CDM, which contains no Met. The pellet was then washed with 2 ml of CDM supplemented with 20 mg/ml L-Met (Sigma) and used to inoculate, at 1/200 dilution, 20 ml of CDM supplemented with 20 mg/ml Met. This culture was grown at 37°C for 16-18 h. Cells were pelleted and washed three times with CDM. Then, the pellet was re-suspended in 20 ml of

CDM supplemented with 20 mg/ml SeMet, which was used to inoculate 1 l of CDM supplemented with SeMet. Cells were grown at 37°C until OD_{600} reached 0.5-0.7. The temperature was then set to 16ºC, the culture allowed to cool for 30 min, before protein expression was induced O/N by adding 1 mM IPTG (Merck) and 4 ml of 36 % glucose (w/v). Two and half hours later, we again added 4 ml of 36 % glucose to the culture. The next day, SeMet-labelled PilB was purified as above.

Crystallisation and structure determination

The PilB crystals used for the high-resolution structure determination were obtained when the purified protein was mixed 1:1 with crystallisation liquor (0.1 M Bis-tris propane pH 7, 3 M NaCl). The lower resolution $PilB_{D319A}$ crystals were obtained with crystallisation liquor (0.1 M Bis-tris pH 6.5, 3 M NaCl). Crystals were cryoprotected with 30 % glycerol in crystallisation liquor, and flash-frozen in liquid nitrogen.

Assaying piliation of *S. sanguinis*

To purify *S. sanguinis* T4P (1, 3), bacteria grown in 100 ml THT until the OD_{600} reached 1-1.5, at which point OD were normalised, bacteria pelleted and re-suspended in pilus buffer (20 mM Tris, pH 7.5, 50 mM NaCl). This suspension was vortexed for 2 min at full speed to shear T4P. After removing bacterial cells by two centrifugation steps and filtration through a 0.22 µm pore size syringe filter (Millipore), pili were pelleted by ultracentrifugation and resuspended in pilus buffer.

Assaying twitching motility of *S. sanguinis*

To assess twitching motility on agar plates (1), bacteria grown O/N were streaked as straight lines on freshly poured TH plates containing 1 % Eiken agar (Eiken Chemicals). Plates were grown for several days at 37ºC in anaerobic condition under high humidity, which is necessary for twitching.

References

1. I. Gurung *et al.*, Functional analysis of an unusual type IV pilus in the Gram-positive *Streptococcus sanguinis*. *Mol. Microbiol.* **99**, 380-392 (2016).

2. I. Gurung, J. L. Berry, A. M. J. Hall, V. Pelicic, Cloning-independent markerless gene editing in *Streptococcus sanguinis*: novel insights in type IV pilus biology. *Nucleic Acids Res.* **45**, e40 (2017).

3. J. L. Berry *et al.*, Global biochemical and structural analysis of the type IV pilus from the Gram-positive bacterium *Streptococcus sanguinis*. *J. Biol. Chem.* **294**, 6796-6808 (2019).

4. R. A. Romijn *et al.*, Identification of the collagen-binding site of the von Willebrand factor A3-domain. *J. Biol. Chem.* **276**, 9985-9991 (2001).

5. S. Kolappan *et al.*, Structure of the *Neisseria meningitidis* type IV pilus. *Nat. Commun.* **7**, 13015 (2016).

6. P. Jones *et al.*, InterProScan 5: genome-scale protein function classification. *Bioinformatics* **30**, 1236-1240 (2014).

Table S1. Strains and plasmids used in this study.

Name	Details	Source
E. coli **strains**		
DH5α	used for cloning	
BL21(DE3)	used for protein expression/purification	
BL21 B834(DE3)	used for SeMet protein expression/purification	
S. sanguinis **strains**		
2908	sequenced WT isolate	(1)
ΔpilB	*ΔpilB::aphA-3* deletion mutant	(1)
ΔpilD	*ΔpilD::aphA-3* deletion mutant	(1)
ΔpilB primary mutant	*ΔpilB::pheS*aphA-3* deletion mutant	this study
pilB$_{D319A}$	*pilB* point mutant expressing PilB$_{D319A}$	this study
**Plasmids**		
pCR8/GW/TOPO	TA cloning vector	Invitrogen
TOPO-*pheS*aphA-3*	*pheS*aphA-3* double cassette in TOPO	(2)
TOPO-*pilB*	*pilB* in TOPO	this study
TOPO-*pilB*$_{D319A}$	*pilB*$_{D319A}$ in TOPO	this study
pET-28b	T7-based expression vector	Novagen
pMK-*pilB*	codon-optimised synthetic *pilB* in pMK	(3)
pET28-*pilB*	pET-28 derivative for expressing 6His-PilB$_{36-461}$	(3)
pET28-*pilB*$_{D319A}$	pET-28 derivative for expressing 6His-PilB$_{D319A}$	this study
pET28-*pilB*$_{vWA}$	pET-2b derivative for expressing 6His-PilB$_{192-461}$	this study

pilB, codon-optimised synthetic gene. *pheS**, point mutant encoding PheS$_{A316G}$.

Table S2. Primers used in this study.

**Name**	**Sequence**
pilB$_{D319A}$ #1	GAAATATATCGTTCTGCTGACCGcTGGTATTCCGAATGCATATCTGG
pilB$_{D319A}$ #2	CCAGATATGCATTCGGAATACCAgCGGTCAGCAGAACGATATATTTC
pilB$_{vWA}$-pET-F	ggg**ccatgg**atcatcatcatcatcatCAGGGCCAGATGAATATTGC
pilB$_{vWA}$-pET-R	ccc**ggatcc**TTACGGACCGCTAACAAACC
pilB-F	TACAACTGGACCGAAGCTGG
pilB-R	TTTGGCCTATCGTTCCCACT
pilB-F1	TACAACTGGACCGAAGCTGG
pilB-R1	gttcttcaatcgttttcgtcatcaTTCCTACCTATTTATTTTTACTTCTG
pilB-F2	ttttactggatgaattgttttagAGGATTTGTGGTTTGTATCAGGG
pilB-R2	TTTGGCCTATCGTTCCCACT
pheS-F*	ATGACGAAAACGATTGAAGAAC
aph-R	CTAAAACAATTCATCCAGTAAAA
pilB$_{D319A}$ *#1*	GCTTAAATATATAGTTCTATTGACAGcTGGCATACCTAATGCTTATTTAGTAG
pilB$_{D319A}$ *#2*	CTACTAAATAAGCATTAGGTATGCCAgCTGTCAATAGAACTATATATTTAAGC

pilB, codon-optimised synthetic gene. Overhangs are in lower case. Restriction sites are in bold. Mismatches are in red.

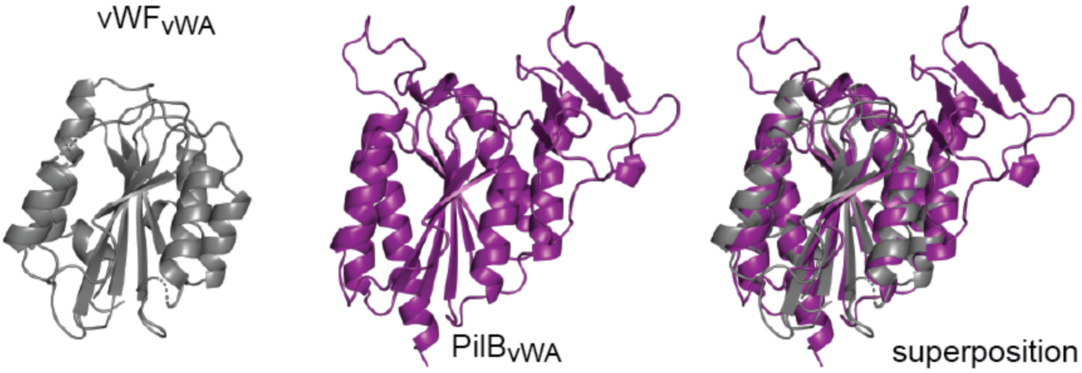

Fig. S1. Structural similarity of vWA domains in PilB and human vWF. Left, A3 vWA domain in vWF (from PDB 1FE8) (grey) (4). Center, vWA module of PilB (purple). Right, superposition of the two structures. While the two sequences share only 15.6 % sequence identity, the structures superpose with an RMSD of 1.72 Å.

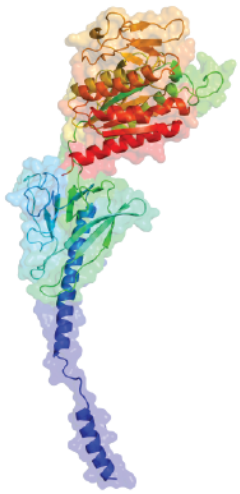

Fig. S2. 3D model of full-length PilB with a melted α1N segment. The cryo-EM structure of the *N. meningitidis* T4P (PDB 5KUA) (5) has been used as a template.

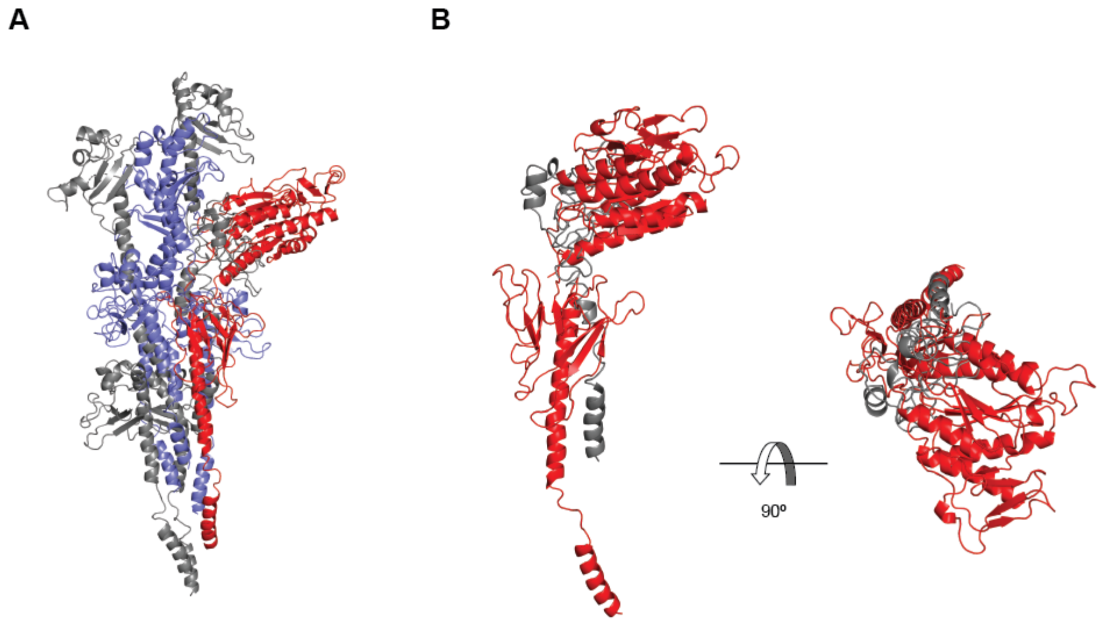

Fig. S3. Modelling of PilB in the body of a T4P leads to important steric clashes. **A**) Packing of PilB (red) into *S. sanguinis* T4P composed of PilE1 (blue) and PilE2 (grey). **B**) Close-up view of the important steric clashes between PilB and PilE subunit above in the filament.

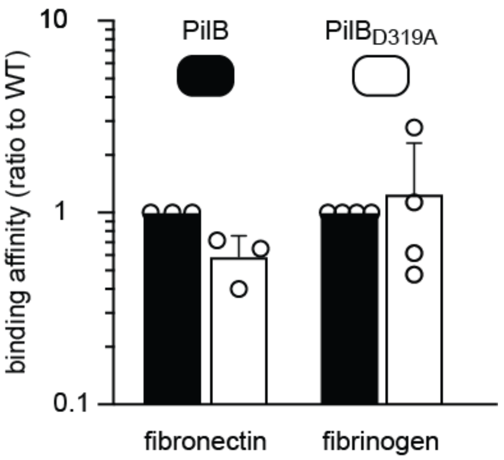

Fig. S4. Binding of $PilB_{D319A}$ to fibrinogen and fibronectin. Increasing concentrations of purified PilB were added to constant concentration of immobilised ligands, and binding was quantified by ELISA. Results are represented as Kd relative to WT, which is set to 1. Results are the average ± standard deviations from 3-4 independent experiments.

Supplementary datasets legends

Supplementary dataset 1. List of all the pilin architectures in the InterPro database. This list was generated by searching the database (November 2020) for entries displaying an IPR012902 pilin motif (6). Modular pilins display an N-terminal pilin motif together with additional module(s) not specific to T4P biology. Proteins in which the pilin motif is not N-terminal are listed as unclear.

Supplementary dataset 2. List of all the PilC/PilY1 architectures in the InterPro database. This list was generated by searching the database (November 2020) for entries displaying the IPR008707 PilC/PilY1 β-propeller domain (6). Modular PilC/PilY1 display a C-terminal IPR008707 motif together with additional module(s) not specific to T4P biology. Proteins in which the IPR008707 motif is not C-terminal are listed as unclear.
